# Editorial: Viruses, innate immunity, and antiviral strategies: from basic research to clinical applications

**DOI:** 10.3389/fcimb.2023.1268363

**Published:** 2023-08-08

**Authors:** Elisa Fanunza, Angela Corona

**Affiliations:** Department of Life and Environmental Sciences, University of Cagliari, Monserrato, Italy

**Keywords:** innate immunity, viral evasion, cGAS/STING, ISG15, HIV, HBV, CHIKV, antivirals

The virus-host interaction is a continuous coevolutionary process. A critical role in the coevolution of viruses and hosts is attributed to innate immunity. Several defense mechanisms have been adopted by the host to clear viral infections, and several other mechanisms of immune evasion have been exploited by viruses (Di Palma et al., 2019; Carty et al., 2021; Fanunza et al., 2021).

This Research Topic highlights the current knowledge on host innate immunity pathways and viral escape and explores the recent advances in antiviral therapeutic strategies.

The induction of the antiviral state of infected cells depends on the recognition of viral components by host pattern-recognition receptors, whose downstream signaling results in the production of interferons and cytokines, leading to the activation of adaptive responses (Carty et al., 2021).

In the cytosol, RIG-I like receptors (RLRs) are the canonical sensors for viral RNA, while the cyclic GMP-AMP synthase (cGAS) is the primary sensor for double-strand DNA (dsDNA). Despite the recognition of viral RNA and DNA relies on diverse receptors, several cross-talk mechanisms between RNA and DNA sensing pathways occur. For instance, the cGAS/stimulator of interferons genes (STING) axis, well known for being activated in response to DNA viral infections, has emerged as RNA sensing pathway in recent years. In the review of Amurri et al. the role of cGAS/STING axis in the control of RNA virus infections has been exhaustively described. The activation of STING has been demonstrated after infection of different RNA viruses. The arising question is: how can these viruses activate the cGAS/STING axis if they do not elicit any DNA intermediate during their life cycle? An indirect activation of STING might be possible, and it may be linked to diverse causes like mitochondrial stress, and chromatin damage in infected cells (Wick et al., 2023). The authors provide a comprehensive overview of how RNA viruses have evolved mechanisms to evade the STING pathway. The first evidence was the discovery of Dengue virus (DENV) non-structural proteins as STING antagonists. This finding was then extended to other Flaviviruses, and other families of viruses, including Coronavirus, Orthomyxovirus, Rhabdovirus, and Togavirus, suggesting the significant contribution of the STING pathway in the protection against RNA viral infections. Due to its importance in viral infection control, STING has emerged as a promising therapeutic antiviral target. In particular, STING agonists have been mainly tested as vaccine adjuvants, potently enhancing antigen-specific antibody production and T-cell responses *in vivo* studies.

The increasing knowledge of innate immune mechanisms has inspired the current challenge of using vaccine adjuvants as a therapeutic strategy against viruses (Schijns et al., 2020). The goal is to build a desired immune response against a specific antigen, ideally with long‐term immunological memory. A suitable immunostimulator is ISG15, an interferon-induced ubiquitin-like protein that plays an important role in the immunity response. Falqui et al. elucidate how ISG15 can induce, prolong, magnify, and steer a specific immune response against the human immunodeficiency virus-1 (HIV-1). An increase in the HIV-1 Env-specific host response occurs after coadministration of DNA vectors codifying for both ISG15 wild type and ISG15 mutant in a heterologous prime-boost combination with a Modified Vaccinia virus Ankara (MVA)-based recombinant vector expressing HIV-1 antigens Env/Gag-Pol-Nef (MVA-B). The authors clearly define the impact of ISG15 on the immunogenicity against HIV-1 antigens in mice, highlighting the importance of ISG15 as an immune adjuvant with potential applicability in therapeutic vaccine approaches against HIV-1.

The lack of a long-term immune system to mount effective immune responses against viruses appears to be critical in the occurrence of Occult hepatitis B virus (HBV) Infection (OBI). Wang et al. describe OBI as the persistence of replication-competent viral genome, a covalently closed circular DNA (cccDNA), in the nuclei of infected hepatocytes and/or in the blood of individuals who test negative for HBV surface antigen (HBsAg). The failure of viral clearance is due to the longevity of the cccDNA and the lack of effectiveness of a host immune response against the virus. Once HBV infection is established, the OBI status can persist even for life. The clinical implications of OBI include the risk of liver cirrhosis and hepatocellular carcinoma, and HBV reactivation or spread through liver transplant or blood transfusion. The OBI prevalence in the population is still largely undefined, with the major limitation being the lack of sensitive and standardized diagnostic assays.

The molecular understanding of the viral-host interface is essential in the research of rewarding therapeutic approaches to control the spread of viral infections. Despite the continuous efforts of the scientific community, a cure remains still an elusive goal for many infections. It is the case for HIV-1. Since HIV-1 appearance 42 years ago, more than 30 antiretroviral drugs have been approved, and combination therapies have transformed HIV-1 infection into a manageable chronic disease. However, drug toxicity and resistance are still the major threats to therapy success. Corona et al. propose a multi-target drug approach in the effort to develop novel HIV-1 inhibitors. This strategy allows the multiple inhibition of the two catalytic activities of the reverse transcriptase (RT), both polymerase and Ribonuclease H (RNase H), and, in addition, the integrase (IN), in an allosteric mode. 4 out of 35 indolinone-base multitarget compounds were able to block the HIV-1 replication in the micromolar range, with compound 10a being the most promising derivative for further drug development.

A drug repurposing approach has been proposed by Kasabe et al. to the aim of identifying novel inhibitors of Chikungunya virus (CHIKV). So far, no licensed drugs or vaccines are available. 9 out of 14 FDA-approved drugs resulted active in blocking CHIKV replication *in vitro*. *In silico* docking analysis suggested that the compounds were potentially able to bind the envelope protein, capsid, and three non-structural proteins. Given the ability of some of these compounds to inhibit also DENV replication, they are very promising for future development and might be used in areas where both viruses are endemic.

In conclusion, the Research Topic recaps some of the most recent progresses into innate immunity, viral evasion and drug discovery, whose understanding proves to be useful in the future for the control and perhaps prevention of viral infections.

## Author contributions

EF: Conceptualization, Writing – original draft, Writing – review & editing. AC: Writing – review & editing.
